# Request a woman scientist: A database for diversifying the public face of science

**DOI:** 10.1371/journal.pbio.3000212

**Published:** 2019-04-23

**Authors:** Elizabeth A. McCullagh, Katarzyna Nowak, Anne Pogoriler, Jessica L. Metcalf, Maryam Zaringhalam, T. Jane Zelikova

**Affiliations:** 1 Department of Physiology and Biophysics, University of Colorado-Anschutz, Aurora, Colorado, United States of America; 2 500 Women Scientists, Boulder, Colorado, United States of America; 3 The Safina Center, Setauket, New York, United States of America; 4 Department of Animal Sciences, Colorado State University, Fort Collins, Colorado, United States of America; 5 Department of Botany, University of Wyoming, Laramie, Wyoming, United States of America

## Abstract

A global online register of women scientists, ready to share their science, was established by a cohort of volunteer women from the grassroots organization 500 Women Scientists on January 17th, 2018. In less than one year, the database “Request a Woman Scientist” comprised over 7,500 women from 174 scientific disciplines and 133 countries. The database is built upon a voluntary questionnaire regarding career stage, degree, scientific discipline, geographic location, and other self-identifying dimensions of representation. The information was visualized using the software platform Tableau, with dropdown menus that help query the database and output a list of names, email addresses, and websites. The biological sciences and women scientists from the United States of America were best represented in the database. A survey of women in the database conducted in November 2018 showed that of 1,278 respondents, 11% had been contacted since signing up for a variety of engagements, including media, peer review, panel participation, educational outreach, and professional/research connections. These engagements resulted in consultations for articles, video chats with students, and speaking opportunities at conferences and events. With improved functionality and marketing, outreach in the global south, and future translation in other languages, this database will further promote the profile and participation of women scientists across society, which in turn will benefit the advancement of science.

## Abstract 2

This Community Page article describes the “Request a Woman Scientist” resource, which was launched in January 2018 through the 500 Women Scientists organization, and constitutes an important step in increasing the diversity of voices represented as scientists in the public and scientific communities.

## Request a Woman Scientist

On January 17th, 2018, 500 Women Scientists (Box 1) launched the Request a Woman Scientist platform (https://500womenscientists.org/request-a-scientist/) to provide an easy to use tool to increase representation of women scientists in the scientific community and public sphere. The idea came from repeated experiences of seeing all men panels (“manels”) and women’s scientific expertise often excluded in the public realm. A 2017 study analyzing colloquium speakers at 50 prestigious universities found that men were invited to give twice as many talks about their research as women [1]. Conference and symposium organizers repeatedly use the same excuses for this imbalance—from “We tried to find a woman to speak on this panel, but we didn’t know any women who work on this topic” to “All the women we asked said no or were unavailable.” The gender gap in scientific forums trickles down into representations of scientists in the media. Women experts are less likely to be cited as sources in news articles about scientific developments, even though they are more engaged in science outreach and communication compared to their male colleagues [2,3]. The underrepresentation of women perpetuates a broader issue in science—expertise relayed to the public does not reflect the true diversity of people and perspectives in science today and across society at large. Furthermore, the lack of visibility of women experts reinforces the idea that science can only be accessed and informed by people who conform to the white male scientist stereotype. These stereotypes influence the next generation’s perceptions of who can practice science. For instance, in a 2018 study, only three in 10 US children drew a woman scientist when asked to draw a picture of what a scientists looks like [4]. That figure, however meager, is a marked improvement from when the surveys and the first study was conducted between in 1966, 1977, and 1983, respectively, when only 1% of children drew women scientists [5].

**Box 1. 500 Women Scientists**

500 Women Scientists is a grassroots organization with the mission to serve society by making science open, inclusive, and accessible. The organization works to transform leadership, diversity, and public engagement in science. Since its inception, 500 Women Scientists has amplified the voices of women scientists in the public sphere (https://goo.gl/kqrZPg). 500 Women Scientists provides resources (https://500womenscientists.org/resources/) to support the success of women in science and works to change perceptions of what a scientist looks like. 500 Women Scientists is organized through groups of women, called pods, who work to achieve the goals of the overall larger organization in their local communities. Currently, there are 292 pods around the world.

To confront the stereotypes of who can be a scientist and to facilitate contact with women scientists, the Request a Woman Scientist platform serves to connect educational institutions, policy makers, members of the public, media, and others with women scientists across scientific disciplines and geographical regions. These women have indicated their willingness to speak with students or the media, consult on a project, sit on a panel, or serve as a conference keynote speaker. The platform helps people find women with the relevant expertise and experience and, at the same time, highlight other areas for inclusion to deepen representation across dimensions of diversity.

## Who is participating in Request a Woman Scientist?

**Fig 1:**
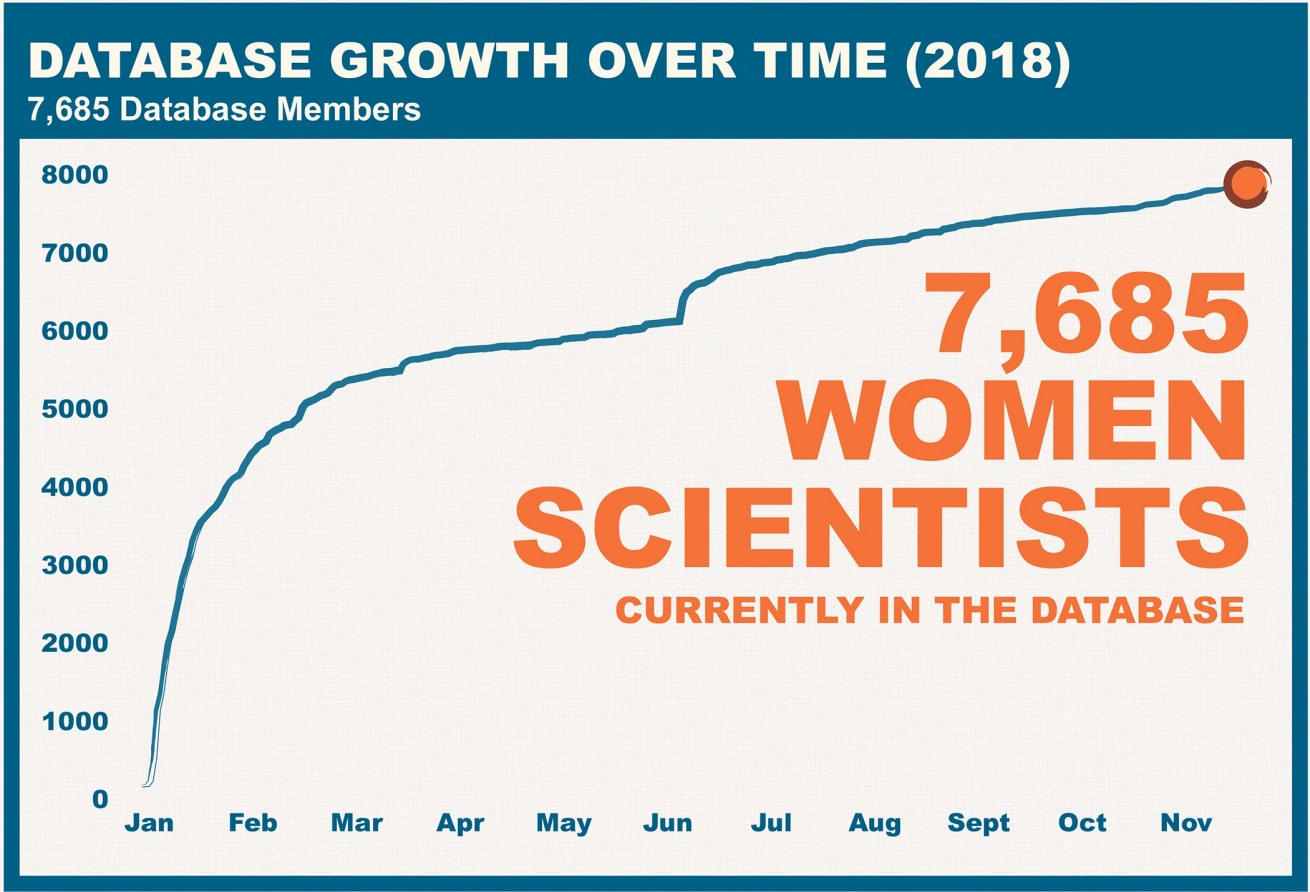
Database growth over time. The number of women who have signed up for the database has grown rapidly in the time since its launch.

As with the 500 Women Scientists open letter (https://500womenscientists.org/our-pledge/) that reached thousands of women across the world, the Request a Woman Scientist platform spread rapidly (sign up here: https://goo.gl/3NJ8hk). Since its launch in January 2018 (Fig 1; all figures were generated in November 2018, and raw data tables are available in S1 Data), over 7,500 women from 133 countries signed up to be a resource, and the platform has been accessed more than 100,000 times by journalists, conference organizers, school teachers, and other scientists.

Self-identified women scientists fill out the Google form and members of 500 Women Scientists vet each entry. This vetting includes checking that the person signing up is indeed a self-identified woman from a science, technology, engineering, math, and medicine (STEMM) field and that the linked information is accurate. The vetting process ensures that the database is accurate and up to date. The searchable graphical user database (gui) was designed in Tableau (Seattle, Washington) as an interactive map with circles of proportional size representing women in that area. The women in the resource are searchable by discipline (which is a set predefined field) as well as by location, participation interest(s) (e.g., keynote address, expert quote, outreach), whether they self-identify as an underrepresented minority, and additional self-defined keywords. The output consists of a list of the names of women scientists, their contact information (most often email address), and their website(s) when provided—the query is exportable as pdf, csv, or weblink.

**Fig 2:**
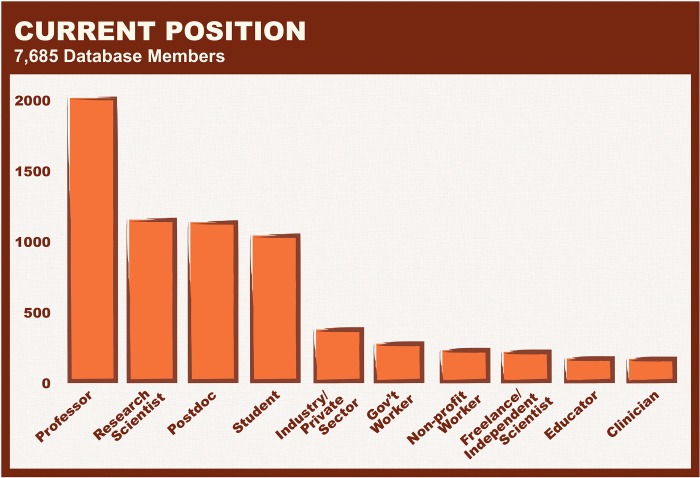
Current position. The number of women represented in each position are shown, with the majority of women in the database identifying as professors.

**Fig 3:**
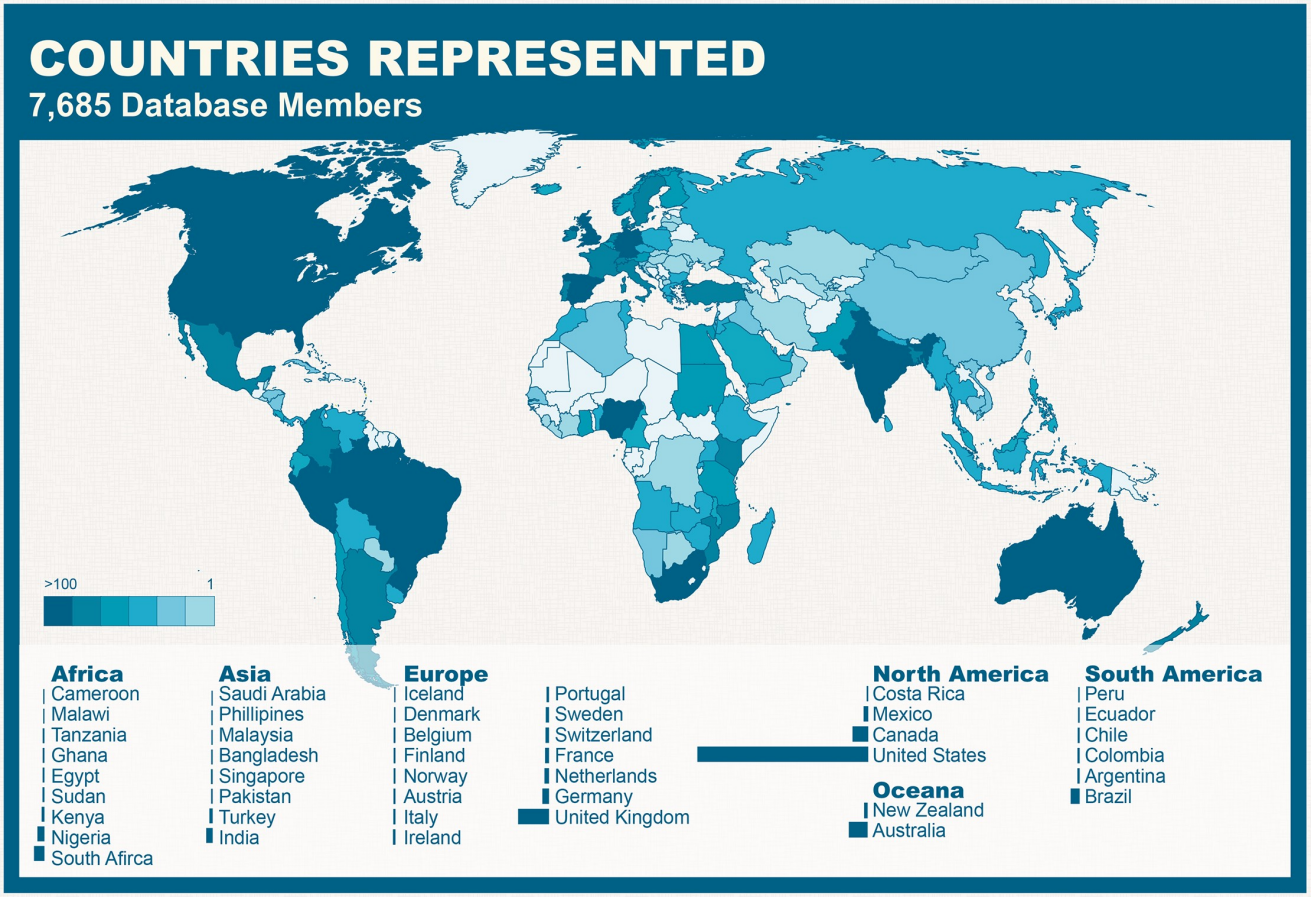
Countries represented. The countries represented in the database are shown in blue, with darker blue indicating greater representation.

Recruitment for the platform has primarily been through social media, with no additional marketing. The resource is composed primarily of faculty members, followed by a roughly equal representation of students, postdocs, and research scientists (Fig 2). Although the platform was launched in the US and approximately half of participants work in the US, the database has had larger than expected international participation, with women scientists from 133 countries represented and highest participation from women scientists in a single location: London, United Kingdom (Fig 3). There are many women participants from almost every continent in the world, with greatest participation in Australia (Melbourne), South Africa (Cape Town), UK (London), India (Bangalore), Brazil (São Paulo), and the US (Washington, DC).

**Fig 4:**
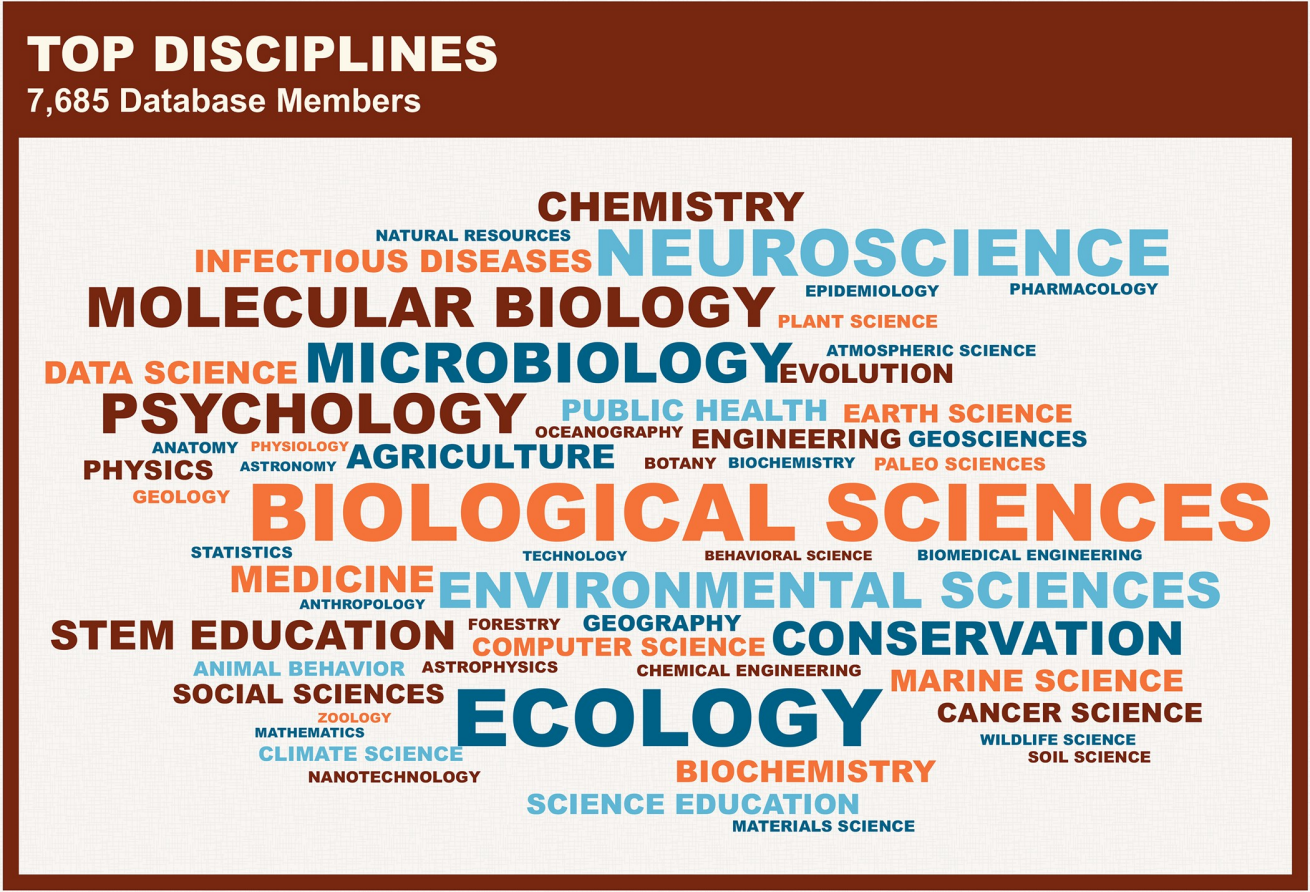
Top disciplines. The top disciplines represented in the database are shown, with size indicating greater representation in that discipline.

Of women who responded, 22.7% (4,338 in total) identify as an underrepresented minority. The majority of women selected biological sciences as their primary discipline (up to three disciplines can be selected) (Fig 4, showing only the primary indicated discipline), which is consistent with data that women are well represented in the biological sciences [6].

## Who is using the Request a Woman Scientist database?

The database was designed to allow users to easily locate women by a number of parameters, including location, field of expertise, degree status, whether they belong to an underrepresented minority group, and through additional custom keywords. The database could be used for multiple purposes. For instance, an organizer for a seminar on climate change could query the resource to find local women climate scientists to invite as speakers. A journalist seeking to find an expert on genetic techniques could query the keyword “CRISPR” (a new method for gene editing) or “gene editing,” while filtering for women interested in speaking to a journalist, to contact their next source. An early career trainee interested in exploring nonacademic careers could use the database to find a woman pursuing a career in policy to contact for an informational interview.

Several journalists have used the database exclusively to source their stories, including for pieces in *The Atlantic* (https://www.theatlantic.com/science/archive/2018/02/i-spent-two-years-trying-to-fix-the-gender-imbalance-in-my-stories/552404/), *Grist* (https://grist.org/article/women-scientists-were-not-backing-down-and-were-not-going-away), and online *National Geographic* (https://news.nationalgeographic.com/2018/02/wildlife-watch-lab-monkey-testing-volkswagen-auto-industry/).

## Who has been contacted through the Request a Woman Scientist database?

**Fig 5:**
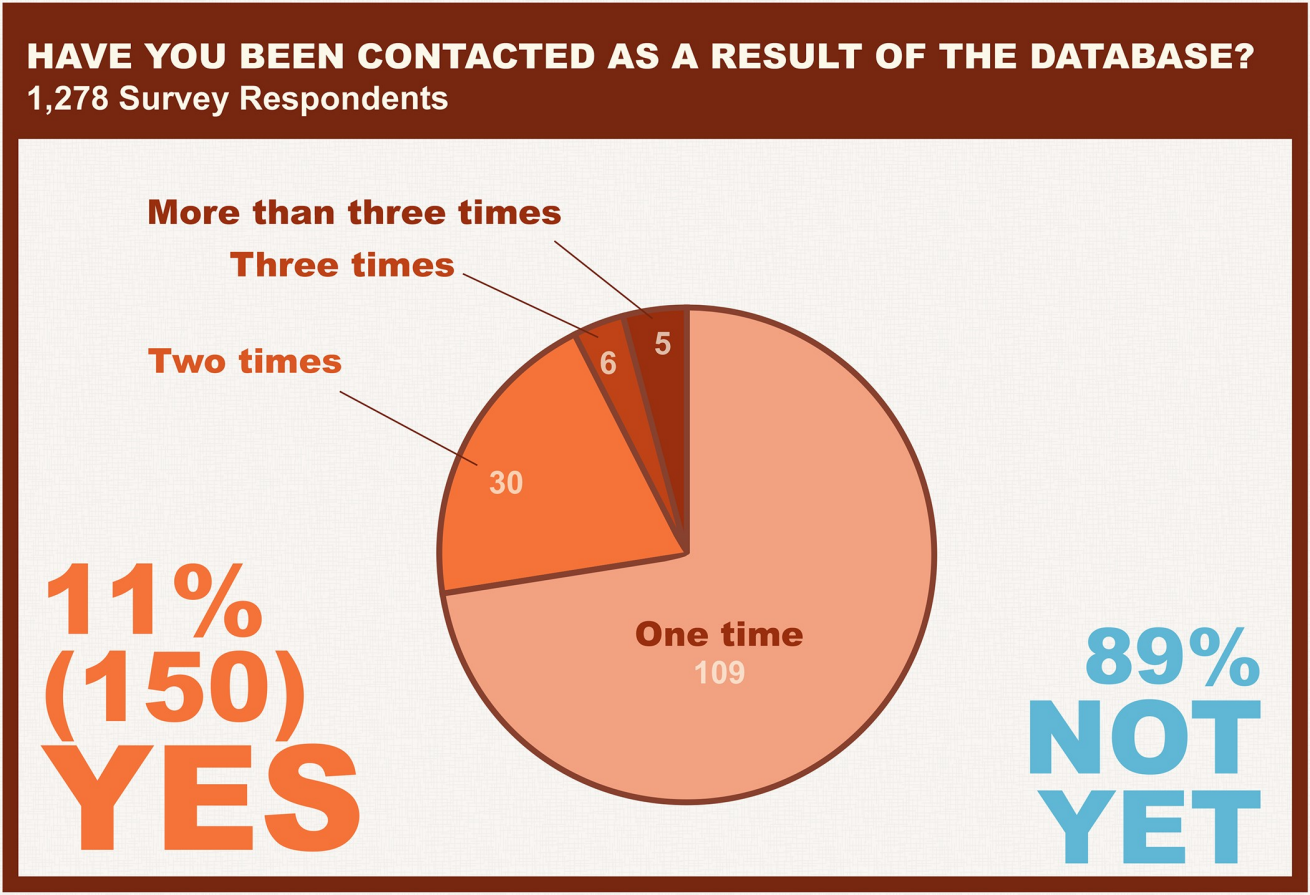
Have you been contacted as a result of the database? Of those who responded to the survey, 11% have been contacted, with the majority of survey respondents having been contacted once.

**Fig 6:**
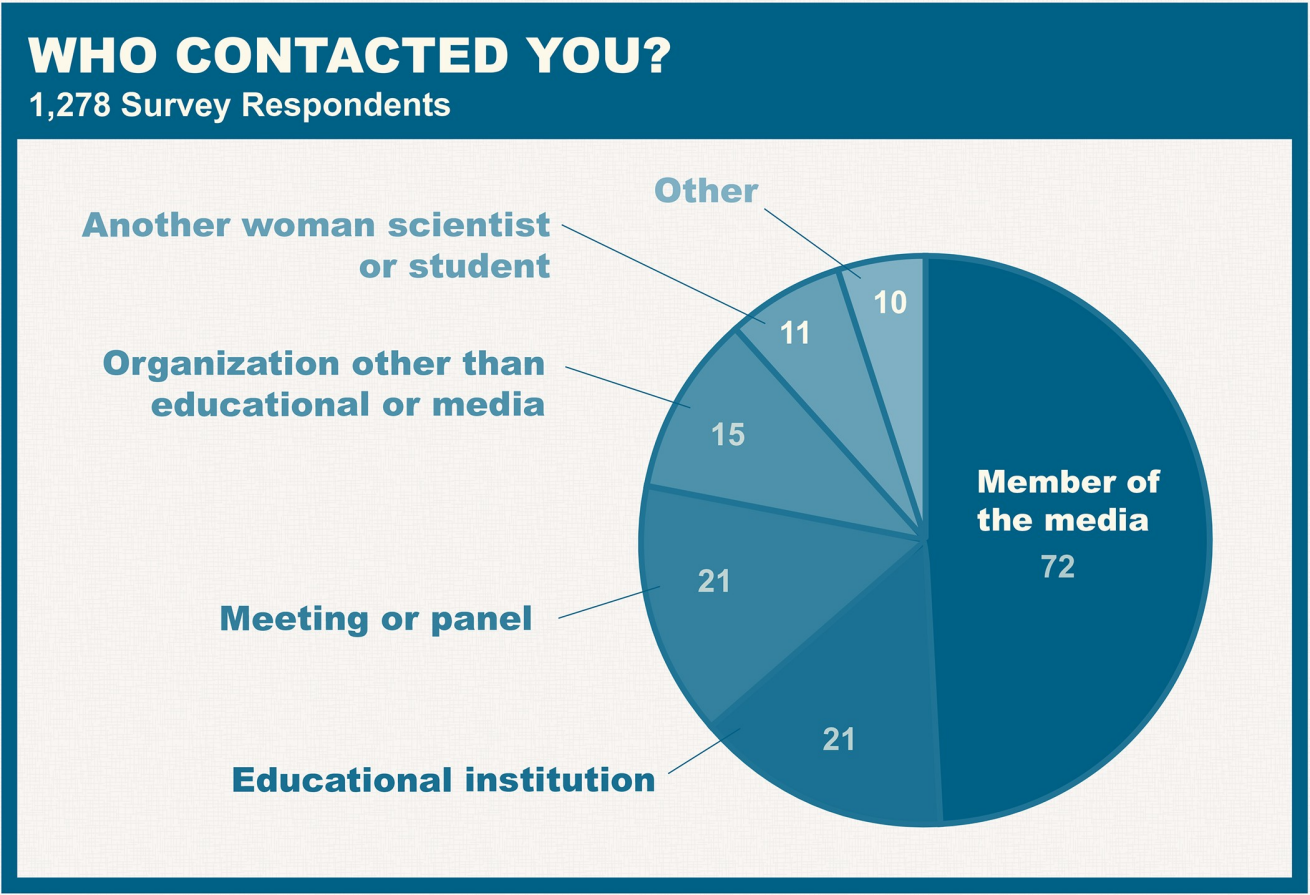
Who contacted you? The majority of those who responded to the survey and had been contacted through the “Request a Woman Scientist” resource were contacted by members of the media.

**Fig 7:**
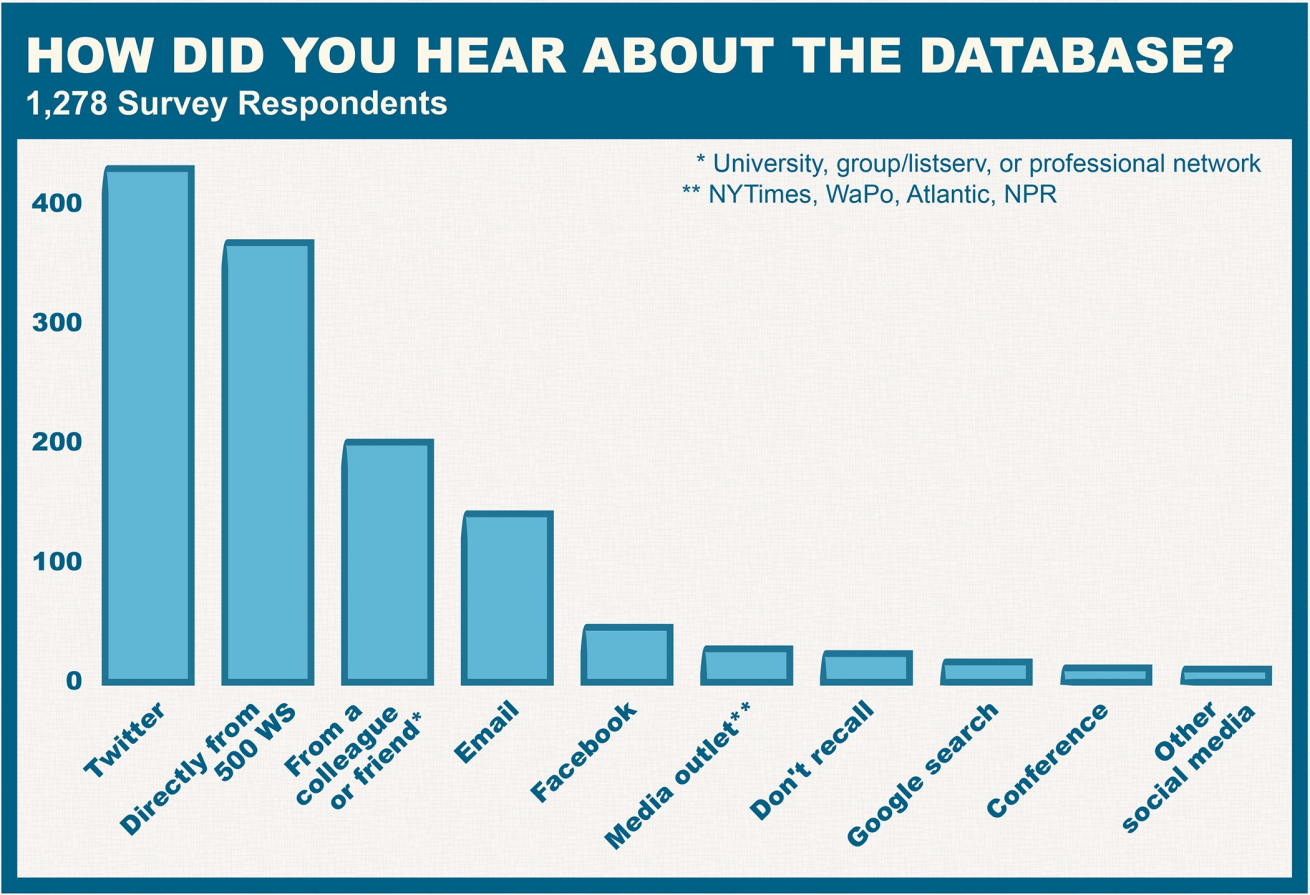
How did you hear about the database? The majority of those who responded to the survey heard about the “Request a Woman Scientist” resource from Twitter.

With a range of possible applications, 500 Women Scientists sought to better understand how the database was being used in practice. In November 2018, a voluntary survey was distributed to all women in the database. Of the 7,500 women surveyed, 1,278 responded (16%). One of the goals of the survey was to assess whether women were being contacted through the resource and by whom. Of those who responded to the survey, 11% (150 individuals) had been contacted through the resource, with the majority contacted only once (Fig 5). This figure may be an underestimate of the true number of women contacted through the resource, as users of the database may not have specified that they found a given expert through the resource. As can be seen in Fig 6, members of the media represent the majority of users of the resource, followed by conference organizers and educational institutions. Lastly, in order to improve future marketing and recruitment of women, women were asked how they heard about the resource, and top responses included Twitter, 500 Women Scientists, colleagues, Facebook, and media coverage (Fig 7).

## Next directions

In 2019, the database will be revamped to improve its functionality and integrate additional disciplines, including the medical sciences. At the same time, the platform will include tracking features to assess how the site is being accessed and to measure impact. The goal of the Request a Woman Scientist platform is to increase representation of women scientists in society and change perceptions of what a scientist looks like. Therefore, building assessment functionality into the platform is critical to understand whether the resource is meeting its intended goals. In addition, the option to save queries for future use will be created by implementing user profiles, which will in turn enable tracking who is using the resource and for what purpose. 500 Women Scientists will also explore promotional strategies to increase enrollment of women from disciplines, industries, and geographical regions that are currently underrepresented in the database, including women in engineering and technology and women from the Global South. A possible approach would be targeting communities through societies like the Institute of Electrical and Electronics Engineers (IEEE), Women in Technology International (WITI), and Organization for Women in Science for the Developing World (OWSD) through strategic partnerships or outreach at conferences.

## Other resources

The Request a Woman Scientist resource has inspired women in medicine to create a similar platform, with fields that are more specific to the medical sciences. 500 Women in Medicine has been created as a subgroup, recognizing that the medical communities have additional unique needs from those of the scientific research community. As a result, 500 Women Scientists is working to build an integrated database that can serve those needs as well as those of the broader STEMM disciplines. In addition, there are other resources that aim to increase participation of underrepresented groups in the sciences or to help scientists engage with the community, a selection of which are highlighted in Table 1.

**Table 1:** Resources related to 500 Women Scientists’ Request a Women Scientist.

Resource name	Purpose	Geographical region	Discipline(s)	URL
Academic.net	Profile excellent female researchers	Europe	All	http://www.academia-net.org/
American Physical Society Minority Speakers List	List names and contact information for underrepresented minorities in physics who are willing to speak on a variety of topics	North America	Physics	https://www.aps.org/programs/minorities/speakers/index.cfm
American Physical Society Women Speakers List	List name and contact information for women physicists who are willing to speak on a variety of topics	North America	Physics	https://www.aps.org/programs/women/speakers/index.cfm
Anne’s List	Highlight female systems neuroscientists	Global	Neuroscience	https://anneslist.net/
The Brussels Binder	Improve diversity in policy debates	Global	Policy	https://brusselsbinder.org/
Climate Voices	Bring together citizens and scientists to discuss climate science and local climate change impacts	Global	Climate Science	https://climatevoices.org/
Diversify EEB	Highlight ecologists and evolutionary biologists who are women and/or underrepresented minorities	Global	Ecology and Evolutionary Biology	https://diversifyeeb.wordpress.com/ (currently migrating to a new site)
Diverse Sources	Make it easy for people to find underrepresented experts in areas of science, health, and environment	Global	Science, Health, Environment	https://diversesources.org/
Diversity Chemistry	Highlight the diverse community of academic chemists	Global	Chemistry	https://diversifychemistry.com/
IWS network	Build a collaborative environment for immigrant and international women in science that nourishes inclusion and diversity in Canada	Canada	All	iwsnetwork.ca
SciLine	Provide timely access to trustworthy, articulate scientific experts for journalists and other communicators working in print, broadcast, or digital fields	Global	SciComm	https://www.sciline.org/
Skype a Scientist	Match scientists with classrooms	Global	All	https://www.skypeascientist.com/
TechWorld’s UK Women in Tech Speaker List	List of women in technology who can speak at tech events	UK	Technology	https://www.techworld.com/careers/here-are-571-uk-women-who-could-speak-at-your-tech-event-3645661/
Women Also Know Stuff	Provide searchable database that promotes and publicizes work and expertise of political scientists	Global	Political Science	https://womenalsoknowstuff.com/
Women in Agriculture	Provide list of women in agriculture	Global	Agriculture	https://docs.google.com/spreadsheets/d/1DbEnbdpjGz1VB28BRlJY9sKBpg0R5bDc7uRb9kfq-DE/edit#gid=0
Women in Astronomy	Provide a database for women in astronomy	Global	Astronomy	https://cswa.aas.org/WIAD.html
Women in Machine Learning	Directory of women in machine learning	Global	Machine Learning	https://wimlworkshop.org/sh_projects/directory/
Women’s Media Center	Raise visibility, viability, and decision-making power of women and girls in media	USA	Media	http://www.womensmediacenter.com/shesource
Women in Microbiology	List of women in fields of microbiology and related fields	Global	Microbiology	https://microbiomedigest.com/sample-page/women-in-microbiology-for-keynote-lectures/
Women in Polar Science	Connect women in polar science	Global	Polar Science	https://twitter.com/womeninpolarsci?lang=en
Women in Soil Science	List of women in soil science and overlapping fields such as agronomy	Global	Soil Science	https://franciskadevries.wordpress.com/women-in-soil-science/
Women in Radiology Informatics	Advance women in medical imaging informatics	Global	Radiology Informatics	http://radxx.ambrahealth.com/community/
Women in Theoretical/Computational Chemistry, Material Science, and Biochemistry	Provide directory of women in theoretical/computational chemistry, material science, and biochemistry	Global	Theoretical/Computational Chemistry, Material Science, and Biochemistry	http://iopenshell.usc.edu/wtc/index.html

## Conclusions

There is a clear need for databases such as Request a Woman Scientist. This is evident from its use by members of the media, educational institutions, researchers, and others. The resource tackles the key excuse of panel organizers of “not being able to find women experts.” However, despite the success of the resource to attract enrollees from around the world in its first year, 500 Women Scientists is well aware that there are far more than 7,500 women in STEMM fields globally. With a revamp of the platform to improve functionality and dedicated marketing, the resource aims to include more women scientists from around the world and to offer an easy-to-use tool to boost visibility of women scientists.

## Supporting information

S1 Data The data used to generate the figures in this manuscript. Each tab indicates the data for each respective figure.
(XLSX)

## Abbreviations

gui: graphical user database
IEEE: Institute of Electrical and Electronics Engineers
OWSD: Organization for Women in Science for the Developing World
STEMM: science, technology, engineering, math, and medicine
WITI: Women in Technology International
